# New perspectives on mobile genetic elements: a paradigm shift for managing the antibiotic resistance crisis

**DOI:** 10.1098/rstb.2020.0462

**Published:** 2022-01-17

**Authors:** Timothy M. Ghaly, Michael R. Gillings

**Affiliations:** ^1^ Department of Biological Sciences, Macquarie University, Sydney, NSW, 2109, Australia; ^2^ ARC Centre of Excellence in Synthetic Biology, Macquarie University, Sydney, NSW, 2109, Australia

**Keywords:** One Health, antimicrobial resistance, horizontal gene transfer, antibiotic pollution, mobile DNA

## Abstract

Mobile genetic elements (MGEs) are primary facilitators in the global spread of antibiotic resistance. Here, we present novel ecological and evolutionary perspectives to understand and manage these elements: as selfish entities that exhibit biological individuality, as pollutants that replicate and as invasive species that thrive under human impact. Importantly, each viewpoint suggests new means to control their activity and spread. When seen as biological individuals, MGEs can be regarded as therapeutic targets in their own right. We highlight promising conjugation-inhibiting compounds that could be administered alongside antibiotic treatment. Viewed as pollutants, sewage treatment methods could be modified to efficiently remove antimicrobials and the resistance genes that they select. Finally, by recognizing the invasive characteristics of MGEs, we might apply strategies developed for the management of invasive species. These include environmental restoration to reduce antimicrobial selection, early detection to help inform appropriate antibiotic usage, and biocontrol strategies that target MGEs, constituting precision antimicrobials. These actions, which embody the One Health approach, target different characteristics of MGEs that are pertinent at the cellular, community, landscape and global levels. The strategies could act on multiple fronts and, together, might provide a more fruitful means to combat the global resistance crisis.

This article is part of the theme issue ‘The secret lives of microbial mobile genetic elements’.

## 1. Introduction

Human activity is the driver of the global antimicrobial resistance crisis, but mobile genetic elements (MGEs) are the primary facilitators [1,2]. To combat antimicrobial resistance, a more complete understanding of their ecology and evolution is needed. MGEs exhibit diversity in function and form. They can be embedded within bacterial chromosomes, such as prophages, insertion sequence elements, transposons and integrative and conjugative elements or can exist as extra-chromosomal molecules, such as plasmids or phage-plasmids [3]. These elements have long been recognized as agents of bacterial evolution and genome innovation by driving the transfer of DNA between different bacterial cells [4]. However, MGEs are now being recognized as more than mere vectors for horizontal gene transfer. Recent research has shown that MGEs have selection pressures and evolutionary trajectories distinct from those of their host cells [5–8]. These distinct selection events result in a dynamic mix of mutualistic and parasitic lifestyles that are adopted by MGEs. Further, plasmid-encoded genes, which are often polyploidic, can be governed by different evolutionary mechanisms from those of their chromosomal counterparts, which usually exist in a haploid state [7].

MGEs have been largely overlooked when considering solutions to curb antimicrobial resistance, despite often being the drivers of its spread [9,10]. In therapeutic contexts, a focus on the bacterial hosts of MGEs has hampered our consideration of MGE ecology and evolution. A shift in mitigation strategies could be helped by adjusting our perception of these elements. This paradigm shift in considering the ecology and evolution of MGEs is an essential step towards managing the antimicrobial resistance crisis. The mechanistic properties of MGEs have been well researched; however, until recently, less attention has been given to the evolutionary and ecological strategies of MGEs. These strategies have important implications for the spread of resistance. For example, plasmid-mediated resistance can persist in the absence of positive selection, even when costly to the host cell [6,8]. Experimental evidence suggests that chromosomal resistance genes only increase in frequency under positive selection, while plasmid-encoded resistance can reach fixation in a population with or without selection [8]. It therefore would be more profitable to consider the resistance crisis from an MGE-centred outlook as opposed to the more traditional host-centric point of view [5].

Here, we present new ecological and evolutionary perspectives from which to view MGEs: as selfish entities that exhibit biological individuality, as pollutants that replicate and as invasive species that thrive under human impact. Each of these viewpoints suggests novel means to control the activity of MGEs and the spread of their clinically important cargo genes.

## 2. New perspectives

### (a) Mobile genetic elements as selfish individuals

Biological individuals can be defined as units that are sufficiently distinct from one another that they can be differentially copied [11]. Units with different rates of replication can be regarded as different individuals. Thus, distinguishing individuals from each other is essential to predict the outcomes of evolutionary processes. In this context, MGEs, which are selfish entities that often have opposing evolutionary strategies to those of their hosts [12], might be more profitably viewed as individual entities, rather than subsidiary components of bacteria.

The presence of MGEs is innately costly to host cells. Fitness costs to the host arise from the processes of conjugation, transposition, plasmid replication or gene expression [5]. Indeed, conjugation, which is induced by the MGE at a cost to the donor cell, can be considered as an example of parasitic manipulation of a host to enhance transmission. Conjugation is a means for self-proliferation of conjugative DNA, with the host bacterium bearing the burden of conjugative protein synthesis, DNA replication and transfer.

In additional to encoding conjugative and replicative proteins that are essential for their horizontal and vertical transmission, plasmids often carry other genes that promote their persistence [7]. These include plasmid partitioning systems, which enhance successful plasmid segregation during cell division [13]; plasmid addiction systems (i.e. toxin-antitoxin genes), which kill any progeny cell that does not inherit the plasmid [14]; anti-restriction genes, which encode DNA-binding proteins that conceal sites targeted by host restriction enzymes [15]; and the recently characterized type IV CRISPR–Cas systems, which are plasmid-encoded defence systems that target other plasmids and are thus believed to be involved in inter-plasmid competition [16]. Plasmids that encode their own extracellular vesicles, facilitating their dissemination and the infection of plasmid-free cells, have also been observed [17], somewhat blurring the distinction between plasmids and viruses.

All of these traits promote the stability, maintenance and successful transmission of MGEs, yet are often disadvantageous to the host cell. Together, they highlight MGEs as biological individuals that are distinct from their host. In a clinical setting, it would be more beneficial to view them as such.

### (b) Mobile genetic elements as pollutants that replicate

Pollution is the dissemination of materials that have harmful effects. MGEs and the antimicrobial resistance genes that they carry have been considered as pollutants [18–21]. While MGEs are natural and ubiquitous components of environmental microbiomes, human activities now release large numbers of these elements in waste streams [22], vastly exceeding natural abundances. Human use of antimicrobials has resulted in the accumulation of MGEs in both human and domesticated animal microbiota, with the consequence that MGEs and antimicrobial resistance determinants are being shed into the environment at rates that overwhelm their abundance in pristine environments [23,24]. A common feature of these polluting DNAs is that they often exist as novel mosaic arrangements that were not present prior to the Industrial Revolution [18,25]. These MGEs, whose assembly and dissemination have been driven by human activity, have been referred to as xenogenetic DNAs [26]. The term ‘xenogenetic’ was coined as an analogue to the term ‘xenobiotic’, which represents compounds synthesized solely by human activity. Likewise, xenogenetic DNAs are the product of the human use of antimicrobials and heavy metals since the Industrial Revolution. Since that time, a series of sequential selection pressures have resulted in a complex amalgamation of MGEs, such as transposons, integrons and plasmids that harbour diverse resistance genes [18,25,27]. Xenogenetic DNAs that harbour various resistance genes are released into the environment from waste streams that also often contain high levels of antibiotics, metals and disinfectants, thus promoting their persistence and widespread dissemination [20,28–30].

An important distinction between xenogenetic DNAs and conventional pollutants is that they can replicate. The concentration of conventional pollutants in the environment decreases with distance from the source of pollution. Further, the longevity of these pollutants is governed by their half-lives. In the case of xenogenetic DNAs, however, their environmental concentrations, spatial distribution and persistence are governed by ecological and evolutionary parameters. This means that a xenogenetic molecule released from a single pollution event at a single point in time and space can dramatically increase in abundance when coupled with the appropriate selection pressures. The global scale of this phenomenon reveals their pervasive and invasive nature.

### (c) Mobile genetic elements as invasive species

The abundance and scale of dissemination of antimicrobial resistance genes have dramatically increased as a direct response to the human use of antimicrobial compounds. These resistance determinants have spread largely via association with MGEs. Antibiotic exposure acts to fix these elements in bacterial populations, while simultaneously inducing their mechanisms of mobility [31,32]. Their spread across phylogenetic boundaries into diverse bacteria and their invasion across vast geographic landscapes [19] bear strong resemblance to invasive species that also spread and thrive under human activity.

Invasive species have successfully spread into alien environments at a global scale, aided significantly by expanding transportation networks [33]. Similarly, humans have changed the transport dynamics of microorganisms via global tourism and trade [22]. Once in a new environment, invasive species thrive in those that are ‘disturbed’ chemically and physically [34]. Likewise, MGEs harbouring antimicrobial resistance determinants thrive and increase in abundance in less pristine environments [35].

Perhaps this is most exemplified by clinical class 1 integrons. These genetic elements, which have played a major role in the acquisition and spread of antimicrobial resistance, most likely originated from a single ancestor within a single cell in the early twentieth century [25,36]. Since that time, derivatives of this ancestral element have spread into more than 70 clinically important bacteria and have been found on every continent [37]. Now, up to 10^23^ copies of these elements are being released from human and agricultural waste every day [22]. This remarkable increase in abundance and dissemination has been driven by antimicrobial selection and global transport.

## 3. From molecular to global scales

**Figure 1.**
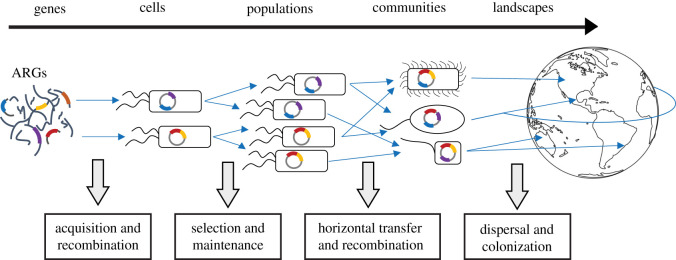
Mobile genetic elements, through recombinatorial and transpositional events, can accrue diverse resistance genes. Due to selection and upregulation in conjugation, genes resident on mobile elements can increase in abundance by vertical and horizontal transmission. This allows their spread into diverse bacteria and their invasion across vast geographic landscapes. The characteristics of mobile elements operate at different scales (top horizontal arrow), ranging from a molecular to a global scale. At each level, we can attempt to interfere with the ecological and evolutionary parameters (bottom text boxes) that facilitate their spread and accumulation of multiple resistance genes. Mitigation strategies that act synergistically on multiple fronts might provide a more fruitful means to combat the global resistance crisis. ARGs, antibiotic resistance genes. (Online version in colour.)

Properties pertaining to each of these viewpoints are evident at the intracellular and extracellular levels and extend to landscape and global scales (figure 1). Integrating these novel perceptions of MGEs might provide us with a more holistic understanding of their ecology and evolution. For example, antimicrobials can drive MGE evolution at the molecular level, resulting in a burst of diversification via the generation of novel, mosaic elements. The subsequent consequences can extend to landscape scales, where novel arrangements of elements can disseminate across the globe, generating further diversity in the process.

A prime example of this is a family of recently characterized mega-plasmids that have disseminated an impressive suite of resistance genes among emerging *Acinetobacter* pathogens [10]. Pangenomic and phylogenomic analyses of these plasmids reveal that they are rapidly spreading across the globe and into new host species, while generating hyper-diversity by acquiring niche-adaptive genes from their local environment. It is clear that such plasmids can amass a different collection of resistance genes depending on the specific antimicrobial selection pressures in their local environments, causing serious concerns for healthcare systems worldwide. In order to tackle the spread of resistance, we need to apply multiple strategies that consider MGE ecology and evolution at all scales.

## 4. Promising applications for reversing resistance

Each of the new viewpoints of MGEs discussed here suggests novel means to control their activity and the spread of their clinically important cargo genes.

### (a) As biological individuals: expanding our therapeutic focus

Perceiving MGEs as units of life separate from their host bacteria allows us to also consider them as separate therapeutic targets. Under antibiotic exposure, genes on MGEs are likely to increase in abundance as a result of upregulated horizontal transmission. As a consequence, antibiotic treatment promotes the diverse pool of resistance genes in the human gut [38] to form new arrangements of multi-resistance elements that then rapidly spread into new bacterial hosts. Thus, during antibiotic treatment, it would be more profitable to expand our therapeutic focus to consider MGEs to prevent the spread of resistance genes. In particular, the use of conjugation-inhibiting compounds alongside antibiotics might help hinder the widespread dissemination of resistance [5,39,40].

Some initial work has shown promise in the search for such compounds. Several pharmaceutical candidates have been discovered to target different components of bacterial conjugation systems. For example, a variety of unsaturated fatty acids are effective conjugative inhibitors in *in vitro* settings [41–44]. It is hypothesized that they inhibit the ATPase activity of the plasmid-encoded TrwD [44], which regulates conjugal pilus biogenesis and DNA translocation [45]. An additional target to inhibit conjugation is the relaxase, a protein involved in conjugation initiation, DNA translocation and final recircularization within the recipient cell. Garcillán-Barcia *et al*. [46] used engineered intracellular antibodies to inhibit conjugation by blocking relaxase activity in recipient cells. Another key target to inhibit conjugation is the type IV secretion pilus, a tubular protein structure necessary for DNA translocation. Several pilus blockers have shown promise, including pilus-binding antibodies, male-specific bacteriophages, Zn^2+^, as well as several chemical compounds, such as chlorpromazine, levallorphan and sodium periodate [40].

A recent study that screened the efficacy of FDA-approved compounds in reducing plasmid transmission has highlighted two nucleoside analogue drugs used to treat HIV, abacavir and azidothymidine [47]. In particular, azidothymidine reduced the transmission of an extended-spectrum β-lactamase-producing plasmid by more than 83.3% in *Escherichia coli* and a carbapenemase-producing plasmid in *Klebsiella pneumoniae* by 80.8%. Importantly, a reduction in plasmid transmission was achieved using azidothymidine concentrations that are attainable in the human gastrointestinal tract. Together, these pharmaceutical candidates are a promising starting platform for *in vivo* safety and efficacy research.

### (b) As pollutants: pollution mitigation

Acknowledging MGEs as novel forms of pollutants is necessary to improve wastewater treatment. Two key components are necessary here. The first is the reduction of bacteria carrying MGEs in wastewater. The second is the removal or degradation of antimicrobials in waste streams that select for these elements.

Several methods to treat sewage are commonly used, however, these methods differ significantly in their ability to reduce antibiotic-resistant bacteria and antibiotic resistance genes [48]. In a comparative analysis, the mechanical-biological (MB) system and MB system with elevated removal of nutrients (MB-ERN) were significantly more efficient in removing resistant bacteria and resistance genes (up to 99.9% removal) compared to the traditional method of anaerobic/anoxic/oxic (A2/O) and sequencing batch reactor (SBR) systems [48]. In particular, SBR and A2/O systems released effluent that was significantly enriched with genes conferring resistance to the new-generation antibiotics cefotaxime and doxycycline.

A number of proposed methods might be suitable for the removal of antibiotics from wastewater. Physical removal of antibiotics from sewage can be achieved using reverse osmosis membranes, which can remove up to 90% of antibiotics from water [49], or by adsorbance by peanut shells, which can remove up to 80% of antibiotics from water. Here, synthetic materials designed to mimic the adsorbent properties of peanut shells might provide a more scalable solution. Such methods might be applied to existing wastewater treatment plants. However, each approach must be scalable to account for the volume of influent per day.

Perhaps the most promising strategy is the use of treatment systems that couple conventional treatment plants with constructed wetlands [50]. This system involves wastewater effluent passing through constructed wetlands. They require no chemical addition and their sludge production is negligible [50]. Instead of concentrating antibiotic pollution, constructed wetlands significantly reduce the loading of antibiotics, antibiotic-resistant bacteria and antibiotic resistance genes [51–53]. The main removal mechanisms include biodegradation, adsorption, precipitation, photolysis and hydrolysis. Increased wetland areas will also have additional ecological, economic and public-use benefits [54–56], although location and land availability might be limiting factors for this strategy. Thus, combining strategies might provide the best approach, particularly for cities with larger influent volumes and limited space.

### (c) As invasive species: invasion management

There is a potential to apply strategies developed for the management of invasive species to control the spread of MGEs [18].

Environmental disturbances are often key drivers that help alien species become invasive. By removing antimicrobial selection in natural environments, we could reduce the advantages of accruing multiple resistance genes, thus preventing the ongoing assembly of novel resistance MGEs. Managing antimicrobial pollution as discussed above is a key step in this strategy. An additional measure is the implementation of regulations and reference environmental limits for antimicrobials, as there are for other pollutants [28]. Such limits will require assessments of no-effect concentrations for diverse selective agents [57]. By restoring natural environments, we could prevent low abundance MGEs from rapidly increasing and becoming invasive.

Early detection of invasive species is a key mitigation strategy [58]. The same strategy could be applied for MGEs. Monitoring and early detection of MGEs and resistance genes can help inform hospital administrations in any given locality about the most appropriate antibiotics to use and those best to avoid. This could improve the efficacy of antibiotic treatment in hospitals as well as preventing favourable selection for MGEs in the local environment.

Biocontrol strategies have also been applied to manage invasive species. In this case, biocontrol agents might be a viable option to kill cells harbouring MGEs of clinical importance without harming other bacteria. This has been successfully achieved already using engineered toxins that selectively target and kill antibiotic-resistant bacteria in mixed populations [59]. In this innovative study, López-Igual *et al*. used toxin genes that were split by inteins and delivered into mixed populations on a plasmid vector via conjugation. The split toxin is only activated in cells that contain specific transcription factors, resulting in cell death. This ‘Trojan Horse’ technique could be tailored to any MGE-specific transcription factor, paving the way for precision antimicrobials. This would soften many of the evolutionary consequences that stem from the blanket approach of broad-spectrum antibiotics. However, such applications must first seriously consider any unintended negative outcomes, which can sometimes greatly outweigh potential positives, as has been the case with hasty biocontrol attempts of agricultural pest species [60]. The release of any replicative agent has the potential for such unintended consequences. For the biocontrol of MGEs, using agents that cannot replicate, such as CRISPR systems or engineered bacteriophage, might provide a safer option.

## 5. Conclusion

The prevalence of pathogenic bacteria that are resistant to one or more antibiotics has been increasing [61], showing that existing attempts to curb antibiotic resistance are failing. Here we argue that MGEs, being the primary facilitators of the spread of resistance, must be considered in mitigation strategies. Importantly, the evolutionary and ecological traits of MGEs should be recognized and used to leverage our attempts to curb resistance. A shift in mitigation strategies could be helped by a shift in our perception of these elements. In particular, MGEs can be viewed as biological individuals, replicating pollutants and as invasive species. Each novel viewpoint highlights particular evolutionary and ecological characteristics of MGEs that are likely to be critical to successfully combat resistance. Further, these characteristics of MGEs might be used to develop promising strategies to control their activity and the dissemination of their clinically important cargo genes. These consist of expanding our therapeutic focus to target the movement of MGEs, improving pollution control and applying invasion management to MGEs.

Importantly, all of the strategies we discuss target different aspects of the ecology and evolution of MGEs, which are pertinent at multiple levels—those being, within a bacterial cell, among microbial communities, across landscapes, and finally, at a global scale (figure 1). By combatting the spread of resistance at each of these fronts, the chance of successfully curbing resistance is much greater.

## Data accessibility

This article has no additional data.
